# Diabetes

**Published:** 1896-05

**Authors:** 


### DIABETES.

Sir George Johnson proposes a new test for urine, in which, to an equal volume of urine and saturated water solution of Picric acid, half its volume of Liquor Potassi is added. An orange red color instantly appears as a result of the incipient reducing action of Picric acid at ordinary temperature. The color is deepened by boiling, and if after about a minute at this temperature a bright red color appears through the test-tube when held up to the light, the urine is free from sugar. A solution containing two grains of glucose to the ounce of water is rendered so dark that no light is visible through the full diameter of the tube. It is asserted by the author that traces of sugar do not exist in normal urine.—*The N. Y. Med. Times.*

